# Comparative Chronic Liver Toxicity of Benzo[*a*]pyrene in Two Populations of the Atlantic Killifish (*Fundulus heteroclitus*) with Different Exposure Histories

**DOI:** 10.1289/ehp.0901799

**Published:** 2010-05-25

**Authors:** Lauren P. Wills, Dawoon Jung, Kara Koehrn, Shiqian Zhu, Kristine L. Willett, David E. Hinton, Richard T. Di Giulio

**Affiliations:** 1 Nicholas School of the Environment, Duke University, Durham, North Carolina, USA; 2 Department of Pharmaceutical and Biomedical Sciences, South Carolina College of Pharmacy, Medical University of South Carolina, Charleston, South Carolina, USA; 3 Department of Physiology, Dartmouth Medical School, Hanover, New Hampshire, USA; 4 Department of Pharmacology and Environmental Toxicology Research Program, University of Mississippi, University, Mississippi, USA

**Keywords:** benzo[a]pyrene, Fundulus heteroclitus, hepatotoxicity, polycyclic aromatic hydrocarbons

## Abstract

**Background:**

The Atlantic Wood Industries Superfund site on the Elizabeth River (ER) in Portsmouth, Virginia, is contaminated with polycyclic aromatic hydrocarbons (PAHs) derived from creosote. Embryos and larvae of ER killifish (*Fundulus heteroclitus*) are refractory to the induction of enzymes regulated by the aryl hydrocarbon receptor including cytochrome P4501A (CYP1A) and are resistant to PAH-induced lethality and teratogenicity. However, adult ER killifish show a greater prevalence of hepatic and pancreatic tumors compared with those from reference sites.

**Objectives:**

We used controlled laboratory studies to determine if ER killifish are more or less sensitive to PAH-induced chronic hepatic toxicity than killifish from an uncontaminated site.

**Methods:**

Larvae from the ER and a reference site on King’s Creek (KC) were subjected to two 24-hr aqueous exposures of benzo[*a*]pyrene (BaP; 0–400 μg/L). At various time points, larvae were analyzed for CYP1A activity, BaP concentrations, nuclear and mitochondrial DNA damage, and liver pathology.

**Results:**

CYP1A activity was induced by BaP in KC but not ER larvae, and KC larvae demonstrated a greater reduction in whole-body concentrations of BaP over time. Mitochondrial and nuclear DNA lesion frequency increased significantly in BaP-exposed KC larvae, but not in ER larvae. Nine months postexposure, KC juveniles exhibited significantly more hepatic foci of cellular alteration and only KC juveniles developed hepatocellular carcinomas.

**Conclusions:**

In addition to acquiring the heritable resistance to the acute teratogenic effects of PAHs, ER fish appear to have concomitantly developed resistance to chronic effects, including cancer.

Atlantic Wood Industries (AWI) on the Elizabeth River (ER) in Portsmouth, Virginia, was a wood-treatment facility from 1926 until 1992. In 1990 the area was classified as a Superfund site because of extensive pollution with the wood preservatives pentachlorophenol (PCP) and creosote (Bieri et al. 1986). Creosote is a complex mixture of unsubstituted, heterocyclic and phenolic polycyclic aromatic hydrocarbons (PAHs), which are by-products of organic combustion. The mean concentration of total PAHs in the AWI sediment is 410 μg/g dry weight; the dominant PAHs include fluoranthene, benzo[*a*]pyrene (BaP), pyrene, and chrysene (Bieri et al. 1986; Vogelbein et al. 2008). The sediment is acutely toxic and highly teratogenic to a variety of species of aquatic organisms including American oysters (*Crassostrea virginica*), brackish water clams (*Rangia cuneata*), and fish such as spot (*Leiostomus xanthurus*), hogchocker (*Trinectes maculates*), and Atlantic killifish (*Fundulus heteroclitus*) (Bender et al. 1988; Hargis et al. 1984; Huggett et al. 1992; Wassenberg and Di Giulio 2004). However, there is a population of reproductively successful killifish that inhabit the contaminated site. Embryos and larvae born to wild-caught ER parents are resistant to the acute toxicity and developmental abnormalities that occur in embryos of reference site adults exposed in the laboratory to the creosote-contaminated sediment (Ownby et al. 2002; Wassenberg and Di Giulio 2004). However, environmentally exposed ER adults exhibit elevated rates of liver cancer (Vogelbein et al. 2008).

PAHs are teratogenic, immunotoxic, and narcotic; however, the carcinogenic effects of PAHs are the most well characterized (Billiard et al. 2008; Carlson et al. 2004; Di Toro and McGrath 2000; Yan 1985). Carcinogenesis requires metabolic activation by enzymes, including the cytochrome P450 (CYP)1 family, which generates DNA adduct-forming products of some PAHs, including the BaP metabolite BaP-7,8-dihydrodiol-9,10-epoxide (BPDE) (Shimada and Fujii-Kuriyama 2004). Expression of genes coding for CYP1 proteins are regulated by the aryl hydrocarbon receptor (AHR), a ligand-activated receptor that binds certain xenobiotics including dioxins, polychlorinated byphenyls (PCBs), and PAHs, leading to marked increases in CYP1 protein levels and enzymatic activities (Denison and Nagy 2003; Hahn et al. 1998). Many of the toxic effects of these compounds are associated with their ability to bind to the AHR (Billiard et al. 2002; Incardona et al. 2006; Prasch et al. 2003).

The ER embryonic, larval, and adult killifish show significantly reduced inducibility of *CYP1A* both at the level of mRNA induction and protein activity compared with those from a reference site (Meyer et al. 2003). This alteration is partially heritable through the F_1_ generation and is thought to play a role in mediating their resistance to teratogenicity and lethality caused by PAHs (Meyer and Di Giulio 2002; Meyer et al. 2002). However, ER killifish larvae are more susceptible to hypoxia and phototoxicity, suggesting that there may be tradeoffs and fitness costs associated with the resistant phenotype (Meyer and Di Giulio 2003). Additionally, adult killifish collected in the vicinity of the AWI site have an elevated incidence of hepatic and extrahepatic lesions compared with those from reference sites (Vogelbein et al. 1990, 2008). Therefore, killifish from the ER population are resistant to the acute and developmental toxicity of PAHs, but their susceptibility to chronic effects, including carcinogenicity, remains unclear.

In this study we tested the hypothesis that although embryonic and larval ER killifish are more resistant to acute PAH toxicity, the adaptation and altered responsiveness of the CYP1 enzymes may have resulted in changes in sensitivity to the genotoxic and carcinogenic effects of PAH exposure. To examine this question, we dosed F_1_ larval killifish born to wild-caught parents from the ER and from King’s Creek (KC; reference site) with a two-hit exposure to BaP. We examined inducibility of CYP1A activity, the time course of BaP biotransformation and excretion, and DNA damage both in the nuclei and in the mitochondria, and we performed histopathological analyses of liver tissue. Our results indicate that in addition to previous reports demonstrating the marked resistance of ER killifish to the acute teratogenic toxicity of PAHs, this population is also relatively resistant to their hallmark chronic hepatotoxicity.

## Materials and Methods

### Fish care

Adult killifish were collected from a reference site at KC in Gloucester County, Virginia (37°17′52.4″N, 76°25′31.4″W), and from a creosote-contaminated site on the ER in Portsmouth, Virginia (36°48′27.48″N, 76°17′35.77″W). Fish were kept at 23–25°C in 25 ppt artificial seawater (ASW) and were maintained on a photoperiod of 14 hr:10 hr light/dark; they were fed Tetramin Tropical Fish Food (Tetra Systems, Blacksburg, VA) and newly hatched brine shrimp (*Artemia*; Brine Shrimp Direct, Ogden, UT). Killifish embryos were obtained from *in vitro* fertilization of pooled oocytes stripped from at least five female fish that were incubated with pooled milt from two or more males. Two hours postfertilization, eggs were treated with 0.3% hydrogen peroxide to prevent fungal infection and then rinsed repeatedly with clean ASW (20 ppt). Eggs developed in petri dishes on damp filter paper and were kept at 23–25°C for 14 days. At 14 days postfertilization, the embryos were hatched by filling the petri dishes with 20 ppt ASW and placed on a shaker for 30 min. Viable larvae were then placed individually into glass scintillation vials and fed a daily diet of newly hatched brine shrimp. Animal care and maintenance protocols were in accordance with regulations mandated by the Duke University Institutional Animal Care and Use Committee. Animals were treated humanely and with regard for alleviation of suffering.

### Chemical exposures

Dimethyl sulfoxide (DMSO), BaP, and ethoxyresorufin were purchased from Sigma-Aldrich (St. Louis, MO). Separate exposures were performed for each of the experimental procedures described. For the *in vitro* ethoxyresorufin-*O*-deethylase (EROD), long amplicon quantitative polymerase chain reaction (LA-QPCR), and histology assays, larvae were dosed in the same manner. Larval killifish from both the ER and KC populations were dosed individually by waterborne exposure 4 days posthatch (dph) to either DMSO (vehicle) or BaP (10–400 μg/L) for 24 hr. Larvae were then placed in clean ASW for 7 days; at 11 dph, larvae were reexposed to the dosing solution for 24 hr. This two-hit dosing regime, adapted from the mouse skin-tumor–promotion model, is based on research that indicates repetitive exposure to mutagenic agents results in the increased accumulation of genetic alterations necessary for tumor development (Dragan et al. 1993; Owens et al. 1999; Tugiyono and Gagnon 2002). DMSO concentrations were maintained at < 0.1% for each treatment.

### EROD assay

Larvae were dosed as described above with either DMSO or BaP (10, 100, or 200 μg/L) for two 24-hr periods 7 days apart. Four days after the second exposure (15 dph), larvae were pooled in groups of 10 (a total of four pools) and homogenized in cold buffer (0.25 M sucrose, 0.1 M tris-HCl, 1 mM EDTA, pH 7.4). The homogenates were centrifuged at 10,000 × *g* for 20 min at 4°C to isolate microsomes. Resultant supernatants were flash-frozen in liquid nitrogen and stored at −80°C. We used the Protein Assay kit (Bio-Rad, Hercules, CA) to determine the protein concentrations of the microsomal preparations.

We measured CYP1A activity using the *in vitro* EROD assay in larval microsomes as described by Willett et al. (1998), with the following modifications. Bovine serum albumin or 200 μg microsomal protein and cofactor buffer (0.1 M Hepes, 100 μM NADH, 115 μM NADPH, and 5 mM magnesium sulfate, pH 8) were loaded into a 96-well plate and incubated at room temperature for 5 min. The addition of 2.5 μM ethoxyresorufin started the reaction. Microsomal enzymatic activity was calculated by determining the production rate of resorufin, the fluorescent by-product of CYP1 metabolism of ethoxyresorufin. Fluorescence was measured at 535/590 excitation/emission each minute for a total of 20 min on a FLUOstar OPTIMA microplate reader (BMG Labtech, Offenburg, Germany). EROD activity is reported as the average picomoles of resorufin per milligram of protein per minute.

### Extractions and chemical analysis

ER and KC larvae were individually dosed on 4 dph with either DMSO or BaP (100 μg/L) for 24 hr. Killifish were frozen in liquid nitrogen 24, 48, or 96 hr after exposure and stored at −80°C until the time of extraction. Larvae were pooled in groups of 10 (a total of four pools), placed in 15 μL methanol/mg tissue, and homogenized according to the protocol described by Hawkins et al. (2002). The resulting homogenate was extracted with 600 μL methanol, filtered through a 0.2-μM nylon Acrodisc (Pall Life Sciences, Ann Arbor, MI), dried under nitrogen, and dissolved in 50 μL acetonitrile. Samples were injected in two 1-μL replicates onto a C-18 reverse-phase ultra pressure liquid chromatography (UPLC) column (ACQUITY UPLC BEH C18, 1.7 μm, 2.1 × 50 mm; Waters Corporation, Milford, MA). We used a three-step gradient elution program to separate the metabolites, as outlined by Zhu et al. (2008). Samples were analyzed by mass spectrometry for the presence of the internal standard 6-OH chrysene, BaP, and the following metabolites of BaP: BaP-3-OH; BaP-9-OH; BaP-1,6-dione; BaP-3,6-dione; BaP-6,12-dione; BaP-7,8-dihydrodiol; BaP-9,10-dihydrodiol; and BaP-7,8,9,10-tetrahydrotetrol. Results are reported as average metabolite concentration calculated by determining the ratio of the metabolite to the concentration of the internal standard recovered.

### LA-QPCR

Larvae were dosed as previously described with either DMSO or BaP (100 or 200 μg/L) for two 24-hr periods 7 days apart. Four days after the second exposure (15 dph), larvae were flash-frozen in 20% glycerol and stored at −80°C. Larvae were homogenized in pools of 10 (a total of four pools), and DNA was extracted using the Genomic-tip 20/G kit (Qiagen Inc., Valencia, CA). LA-QPCR was performed according to a protocol described by Jung et al. (2009). We amplified 10 ng DNA from each sample with *rTth* polymerase (Applied Biosystems, Foster City, CA). Primers for the nuclear targets were designed for the cystic fibrosis transmembrane conductance regulator gene (Jung et al. 2009). The primer sequences for the large mitochondrial target were obtained from Kim et al. (2004). Primers and amplicon sizes were 11.5 kb for nuclear DNA and 9.4 kb for mitochondrial DNA. DNA was amplified, and the resulting concentrations were converted to relative lesion frequencies per 10 kb DNA, based on alterations in amplification efficiency (Ayala-Torres et al. 2000).

### Histology

Larvae were dosed as previously described with either DMSO or BaP (50, 100, 200, or 400 μg/L) for two 24-hr periods 7 days apart (*n* = 60 larvae per treatment group). Subsets of 25–30 juvenile killifish from each treatment were weighed and sacrificed 3 or 9 months after exposure using MS-222 (tricaine methanesulfonate, 100 ppm). Tails were surgically removed, and a midventral incision through the abdominal body wall was extended from the anal pore to the level of the pectoral fins. Individuals were immediately placed in 10 volumes of 10% neutral buffered formalin, decalcified using decalcifying solution (Richard-Allan Scientific, Kalamazoo, MI), dehydrated in a graded ethanol series, cleared in xylene, and embedded in paraffin. Tissue blocks were sectioned at 5 μm, mounted on glass histoslides, and stained with Harris’ hematoxylin and eosin (H&E). For each individual fish we evaluated three sections 10 μm apart with a Nikon Eclipse E600 light microscope equipped with a Nikon DXM 1200 digital camera and EclipseNet imaging software (Nikon, Melville, NY). Analysis was focused on the liver, but the intestine and neighboring mesentery with exocrine pancreas were included within the sections. After the initial read by two separate individuals, concurrence on lesions was established, and the number of positive individuals was determined per treatment.

Tissue aberrations classified as lesions included eosinophilic and basophilic foci of cellular alteration (FCA), hepatocellular adenomas, and early hepatocellular carcinomas as defined by Baumann et al. (1990) and Vogelbein et al. (1990). FCA were defined as foci of altered H&E staining and were basophilic or eosinophilic compared with the surrounding, noninvolved parenchyma. Hepatocellular adenomas were classified as hypertrophied hepatocytes with distinct margins. Hepatocellular carcinomas exhibited disorganized cellular structure and irregular borders, with neoplastic cells invading the surrounding parenchyma. The cytoplasm of hepatocytes within resultant tumors was eosinophilic and fibrillar in nature.

### Statistical analyses

We analyzed data using SPSS Version 15 (SPSS, Inc., Chicago, IL). Data sets were analyzed initially by the Kolmogrov-Smirnov test to determine if they were normally distributed. EROD, chemical analysis, and DNA lesion data were all determined to be normally distributed and were analyzed using analysis of variance (ANOVA). Pairwise comparisons were analyzed for statistical significance using a Bonferroni-corrected post hoc comparison. The lesion data were analyzed using a global chi-square analysis with manual post hoc testing between comparable treatment groups. Statistical significance was accepted at *p* ≤ 0.05 for all tests.

## Results

**Figure 1:**
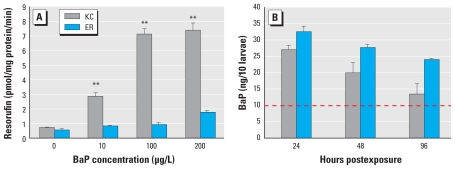
Effect of BaP in KC and ER killifish larvae after laboratory exposure to either the DMSO vehicle (control) or BaP (10–200 μg/L). (*A*) Induction of CYP1 enzymatic activity measured in larvae by the *in vitro* EROD assay 4 days after repeated 24-hr BaP exposures. Data are mean ± SEM; *n* = 4 pools of 10 larvae per treatment group. (*B*) BaP extracted and identified by UPLC/MS 24, 48, and 96 hr after 24 hr exposure to 100 μg/L BaP. The dashed line represents the detection limit. Effects of population, treatment, and time on the recovery of BaP were significant (*p* < 0.05; ANOVA), with more BaP recovered from the ER larvae than from KC larvae. The interaction of population and time was significant (*p* < 0.001), with the KC larvae showing a greater reduction in whole-body concentration of BaP over time than the ER larvae. No BaP was detected in the vehicle control larvae. Data are mean concentration of BaP recovered per 10 larvae ± SEM; *n* = 5 pools of 10 larvae per treatment group. **p < 0.001 compared with control by Bonferroni-corrected ANOVA.

Consistent with previous studies, laboratory-reared larval killifish from parents of the KC population exhibited significant induction of CYP1A enzymatic activity 4 days after repeated 24-hr exposures to 10–200 μg/L BaP (*p* < 0.001) (Figure 1A), whereas ER killifish exhibited no significant induction.

Over 96 hr, the KC larvae showed a greater reduction in whole-body concentrations of BaP over time compared with ER larvae (*p* < 0.001). Chemical analysis revealed a significant interaction between population, treatment, and time for the amount of BaP recovered from the larvae (*p* < 0.05) (Figure 1B). No detectable levels of BaP were recovered from KC or ER DMSO (vehicle control) larvae (data not shown). In KC and ER larvae exposed to 100 μg/L BaP, we observed a significant decrease over time in the amount of BaP recovered from KC larvae (*p* < 0.05) but no significant change in BaP recovered in exposed ER larvae. Recovery of all of the other metabolites (BaP-7,8,9,10-tetrahydrotetrol; BaP-7,8-dihydrodiol; BaP-9,10-dihydrodiol; BaP-1,6-dione; BaP-3,6-dione; BaP-6,12-dione; BaP-9OH; and BaP-3-OH) remained below detection limits; therefore, no conclusions could be made concerning their production in the larvae.

**Figure 2:**
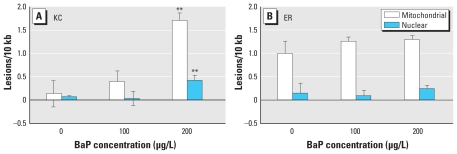
Mitochondrial and nuclear DNA damage in killifish larvae 4 days after repeated 24-hr exposures to DMSO vehicle or BaP (100 or 200 μg/L). Frequency of lesions in (*A*) KC larvae and (*B*) ER larvae. KC larvae showed a significant increase in relative lesion frequency in both mitochondrial and nuclear DNA after exposure to 200 μg/L BaP (*p* ≤ 0.001). The ER larvae showed no significant increase in either mitochondrial or nuclear DNA lesion frequency for any of the concentrations of BaP examined; ER vehicle controls showed a significantly higher level of mitochondrial DNA lesion frequency than KC larvae (*p* < 0.05). Data are mean lesion frequency ± SEM; *n* = 4 pools of two larvae per treatment group. **p < 0.001 compared with control by Bonferroni-corrected ANOVA.

Using LA-QPCR we detected a significant increase in lesion frequency in both nuclear and mitochondrial DNA in KC larvae 4 days after repeated 24-hr exposure to 200 μg/L BaP (*p* < 0.001) (Figure 2A). This increased DNA damage was significantly higher in mitochondrial DNA than in nuclear DNA (*p* < 0.001), but we observed no significant increase in DNA damage in KC larvae after exposure to 100 μg/L BaP. ER larvae had a higher basal level of mitochondrial lesion frequency; however, no significant change in DNA damage was observed after exposure to either 100 or 200 μg/L BaP (Figure 2B).

Mortality throughout the 9-month experiment was < 20% for all treatment groups, and we found no significant interactions between mortality and treatment (data not shown). Similarly, no significant differences in body weight between populations or treatments were observed at either 3 or 9 months postexposure (data not shown).

**Figure 3:**
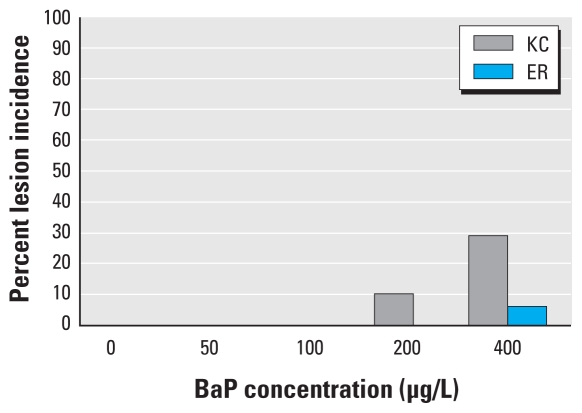
Prevalence of hepatic lesions in KC and ER juvenile killifish 9 months after exposure to BaP (50–400 μg/L). Hepatocellular lesions were diagnosed as FCA, hepatocellular adenomas, and hepatocellular carcinomas, as described by Baumann et al. (1990) and Vogelbein et al. (1990). We observed a significant increase in the incidence of hepatic lesions in KC juveniles exposed to BaP (*p* < 0.05). ER fish showed no incidence of hepatic lesions after exposure to 200 μg/L. Lesion incidence after the 400 μg/L exposure was 20% higher in KC juveniles than in ER juveniles (*p* < 0.05). Data are percent lesion incidence; *n* ≥ 20 juveniles per treatment group.

**Figure 4:**
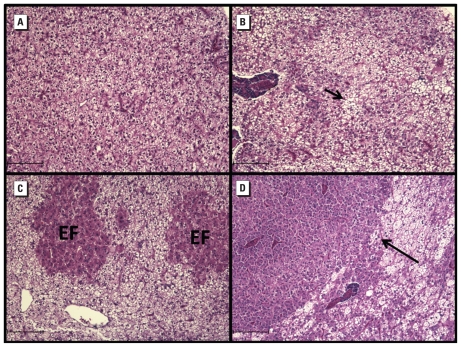
Photomicrographs H&E-stained paraffin sections showing liver pathology (FCA) in KC killifish juveniles 9 months after exposure to DMSO (vehicle control; *A*) or 400 μg/L BaP (*B–D*). (*A*) Normal liver from a vehicle control. (*B*) Focal steatosis in liver from a BaP-exposed killifish; the arrow indicates large rounded vacuoles with smooth margins signifying lipid. (*C*) Eosinophilic foci (EF) in liver from a BaP-exposed killifish. (*D*) Hepatocellular carcinoma in liver from a BaP-exposed killifish; the arrow indicates an irregular and invasive border of the carcinoma. Bars = 100 μm.

We observed no FCA, adenomas, or hepatic neoplasms in KC or ER larvae exposed to DMSO, and no lesions were found in any juveniles 3 months after exposure to BaP (50, 100, 200, or 400 μg/L) (data not shown). Nine months after dosing, 2 of the 25 KC juveniles exposed to 200 μg/L BaP developed eosinophilic FCA, denoting an 8% incidence in lesion frequency; however, this was not significantly different from DMSO controls (Figure 3). Of the 30 KC juveniles exposed to 400 μg/L BaP, 9 developed increased FCA (Figure 3); of these 9 juveniles, 2 also developed early hepatocellular adenomas, and 1 developed a hepatocellular carcinoma (Figure 4D). These observations denoted a 30% incidence in lesion frequency that was significantly different from the DMSO control (*p* = 0.026). Only 2 of the 30 ER juveniles dosed with 400 μg/L BaP developed eosinophilic FCA, resulting in a 6% incidence in lesion frequency; this was not significantly different from controls. KC juveniles exposed to 400 μg/L BaP exhibited a significantly greater lesion frequency than similarly exposed ER juveniles (*p* = 0.018). No hepatocellular adenomas or carcinomas were found in the ER juveniles that we examined.

## Discussion

We compared the genotoxic effects of a carcinogenic PAH, BaP, in offspring of two populations of Atlantic killifish: one from a reference site and one from an area along the ER that is highly contaminated with PAHs. ER embryos and larvae are recalcitrant to *CYP1* induction by AHR agonists and resistant to acute PAH toxicity (Meyer et al. 2003; Wills et al. 2009). However, this population does display multiple fitness costs, including susceptibility as adults to hepatocellular and pancreatic carcinomas (Vogelbein et al. 1999). These findings led us to question whether the offspring of the ER killifish population are more or less resistant than are fish from the reference site (KC) to the chronic toxicity of PAHs.

Consistent with previous studies, BaP exposure caused a significant induction of EROD activity in KC larvae, but not in ER larvae (Meyer et al. 2003; Van Veld et al. 1997). In another study we observed that the refractory phenotype of the ER larvae extends beyond *CYP1A* and is also characterized by decreased mRNA induction of *CYP1B1* and *CYP1C1* (Wills et al. 2010). The differences between the KC and ER killifish in the induction and activity of the CYP1 metabolic enzymes suggest that there are likely to be differences between these two populations in their ability to biotransform BaP either by activation or elimination of the parent compound.

Perhaps because of limited tissue mass, we were not able to detect any of the metabolites of BaP in either population. However, there was a significantly lower amount of the parent compound recovered from exposed ER juveniles compared with those from the KC, indicating that these fish may metabolize BaP at a slower rate than KC fish. Although this reduction in PAH elimination may protect the ER juveniles from carcinogenesis, the adaptations underlying this protection may be associated with the previously reported fitness costs exhibited by ER killifish of increased sensitivity to PAH-induced phototoxicity and hypoxia (Meyer and Di Giulio 2003). Another possibility is that the ER fish have shifted their metabolism toward a less-toxic metabolite that we were not able to detect. In a previous study (Wills et al. 2009), we recovered a significantly higher concentration of the benign metabolite BaP-9,10-dihydriodiol from ER embryos exposed to BaP compared with embryos from KC. ER killifish may have shifted away from the production of other toxic metabolites, including BaP-7,8-dihydrodiol, which is a precursor for the mutagenic BPDE. One possibility for why we were not able to detect BaP-9,10-dihydrodiol in this study is that we were examining larvae as opposed to embryos, which may have different rates of metabolism and excretion. Perhaps analysis of bile from larger juveniles or adults would provide additional information (Malins et al. 1988).

The induction of CYP1A enzymatic activity and the decreased amount of BaP recovered from the KC larvae over time suggest that there was metabolic activity occurring and that BaP was being either biotransformed or excreted. Reactive metabolites of BaP can result in DNA damage through the formation of bulky adducts and through reactive oxygen species (Burdick et al. 2003; Varanasi et al. 1986). In experiments in the present study, we used LA-QPCR to examine general mitochondrial and nuclear DNA injury, including the formation of bulky adducts and oxidative damage, both of which may be playing a role in BaP-induced carcinogenicity. In the KC killifish we observed an increased level of DNA damage after exposure to 200 μg/L BaP compared with the controls. We also observed a higher level of damage in mitochondrial DNA relative to nuclear DNA. This finding is consistent with results in previous mammalian cell culture studies, which indicated that mitochondrial DNA is more susceptible to PAH-induced damage than is nuclear DNA (Allen and Coombs 1980; Backer and Weinstein 1980). We observed that the ER killifish had a higher level of basal mitochondrial DNA damage than larvae from the KC population, indicating that perhaps there is maternal transfer of BaP due to the slow metabolism of the compounds in this population. However, the ER larvae showed no increase in either mitochondrial or nuclear DNA damage after BaP exposure. Nacci et al. (2002) obtained similar results in studies of a killifish population in New Bedford Harbor resistant to dioxin-like compounds. After exposure to BaP, the New Bedford Harbor killifish had lower levels of EROD induction and DNA adduct formation compared with reference site fish, indicating that they may also be resistant to the carcinogenic effects of PAHs.

Epizootics of liver neoplasms in marine and freshwater fish have been associated with carcinogenic PAHs in the sediments of many contaminated waterways, and tumor incidences in wild populations have been used to monitor ecosystem health (Baumann and Harshbarger 1998; Myers et al. 1990; Vogelbein and Unger 2006). Vogelbein et al. (2008) reported that ER killifish had a 93% incidence of liver lesions and a 33% frequency of liver cancer, whereas a reference site population had no detectable lesions. In the present study, offspring of KC killifish exposed to 400 μg/L BaP had a significantly elevated incidence of altered eosinophilic and basophilic foci, hepatocellular adenomas, and hepatocellular carcinomas. Although two ER juveniles developed altered foci, no hepatocellular adenomas or carcinomas were observed, suggesting that ER larvae are resistant to the carcinogenic effects of BaP. Additionally, these lesions were observed only at the highest dose of BaP (400 μg/L), and no altered foci were observed in any of the other BaP treatments (50–200 μg/L).

One reason the offspring of ER killifish may be more resistant to the onset of the carcinogenic effects of BaP is the lack of inducibility of their CYP1 enzymes. Enzymatic modulation of PAH-induced carcinogenesis has been observed in rainbow trout (*Oncorhynchus mykiss*) (Shelton et al. 1984; Takahashi et al. 1996). Additionally, blocking enzymatic activity by knocking down or knocking out the AHR has been shown to provide protection against PAH-induced teratogenicity and carcinogenicity. Shimizu et al. (2000) and Talaska et al. (2006) found that compared with the wild type, the Ahr^−/−^ mice did not develop subcutaneous or skin tumors in response to BaP exposure and had a 90% reduction in BaP adduct levels. Fan et al. (2010) showed that Ahr^−/−^ mice exposed to diethylnitrosamine had an elevated incidence of liver tumor formation compared with the wild type. Diethylnitrosamine is a potent hepatic carcinogen but is not an agonist for the AHR. These data suggest that in the absence of a xenobiotic ligand, the *Ahr* gene can function as a tumor suppressor gene. An interesting possibility for future experiments would be to examine the differences in tumor formation between ER and reference killifish after exposure to a hepatic carcinogen that is not an AHR ligand.

The high prevalence of hepatic lesions in environmentally exposed ER fish is not surprising, given the amounts of carcinogens present in ER sediment and the chronic exposure that this population receives. However, our data suggest that ER killifish are less susceptible to the genotoxic and carcinogenic effects of laboratory exposure to BaP. To determine if the resistance to BaP-mediated chronic liver injury observed in ER killifish in this study extends to the complex chemical mixture present in ER sediments, future studies will include chronic exposures of KC and ER offspring to ER sediment extracts. Although the conclusions drawn pertain directly to killifish, the findings have implications for PAH biotransformation and resultant chronic toxicity in other species and shed further light on vertebrate adaptations to chronic pollution.
